# BoltzNCE: Learning Likelihoods for Boltzmann Generation with Stochastic Interpolants and Noise Contrastive Estimation

**Published:** 2025-07-02

**Authors:** Rishal Aggarwal, Jacky Chen, Nicholas M. Boffi, David Ryan Koes

## Abstract

Efficient sampling from the Boltzmann distribution defined by an energy function is a key challenge in modeling physical systems such as molecules. Boltzmann Generators tackle this by leveraging Continuous Normalizing Flows that transform a simple prior into a distribution that can be reweighted to match the Boltzmann distribution using sample likelihoods. However, obtaining likelihoods requires computing costly Jacobians during integration, making it impractical for large molecular systems. To overcome this, we propose learning the likelihood of the generated distribution via an energy-based model trained with noise contrastive estimation and score matching. By using stochastic interpolants to anneal between the prior and generated distributions, we combine both the objective functions to efficiently learn the density function. On the alanine dipeptide system, we demonstrate that our method yields free energy profiles and energy distributions comparable to those obtained with exact likelihoods. Additionally, we show that free energy differences between metastable states can be estimated accurately with orders-of-magnitude speedup.

### 1 Introduction

Obtaining the equilibrium distribution of molecular conformations, the geometric arrangements of atoms in a molecule, defined by an energy function is a fundamental yet challenging problem in the physical sciences [1–3]. The Boltzmann distribution describes the probability density induced by an energy function and is given by $p(x) \propto \exp(-U(x)/K_B T)$ where $U(x)$ is the energy of molecular conformer $x$, $K_B$ is the Boltzmann constant and $T$ is temperature. Traditional approaches for sampling conformers, such as Markov Chain Monte Carlo (MCMC), and Molecular Dynamics (MD) simulations often get trapped in energy wells, requiring long simulation timescales to produce uncorrelated samples. Consequently, it is particularly inefficient to obtain samples from independent metastable states — a limitation commonly known as the sampling problem.

Preprint. Under review.

In recent years, several generative deep learning methods have been developed to address the sampling problem. One such class of methods is known as Boltzmann Generators [4–7]. These models work by transforming a simple prior distribution (such as a multivariate Gaussian) into a distribution over molecular conformers, which can then be reweighted to approximate the Boltzmann distribution. When the generative model does not involve reweighting, it is referred to as a Boltzmann Emulator [5]. The main goal of a Boltzmann Emulator is to efficiently sample from the metastable states of the molecular ensemble.

To compute the likelihoods of generated samples, Boltzmann Generators are constrained to the class of normalizing flows. While earlier methods built these flows using invertible neural networks [4, 8], more recent approaches prefer using continuous normalizing flows (CNFs) [5, 9] due to enhanced expressitivity and flexibility in model design.

However, computing likelihoods for CNF-generated samples requires expensive Jacobian trace evaluations along the integration path [10, 11]. This computational overhead limits their scalability, particularly for large, full-scale protein systems. In this work, we explore whether these likelihoods can be efficiently approximated by a separate model to avoid the Jacobian trace path integral.

We investigate the use of Energy-Based Models (EBMs) as a means to learn likelihoods. EBMs model the density function as being proportional to the exponential of the predicted energy, i.e., $p_\theta(x) \propto \exp(E_\theta(x))$ [12]. However, scalable training of EBMs remains a major challenge due to the need for sampling from the model distribution, which often requires simulation during training [13, 14]. Therefore, developing efficient training algorithms for EBMs continues to be an active area of research [12, 15–17].

We adopt Noise Contrastive Estimation (NCE) as a training strategy for Energy-Based Models (EBMs) [18]. NCE trains a classifier to distinguish between samples drawn from the target data distribution and those from a carefully chosen noise distribution. A key advantage of this approach is that it circumvents the need to compute intractable normalizing constants [19, 20]. However, NCE can suffer from the *density-chasm* problem [21] that leads to flat optimization landscapes when the data and noise distributions differ significantly, i.e, when the KL divergence between them is large [22].

We tackle this issue by annealing between a simple noise distribution and the data distribution using stochastic interpolants [23, 24]. We further enhance the training process by incorporating an InfoNCE [25] loss along with the score matching objective defined on stochastic interpolants. We show that training with both loss functions shows significant performance improvement over using either individually. Notably, our proposed method for training the EBM is *simulation-free*, avoids the computation of normalizing constants, and is therefore scalable to large systems.

To summarize, the main contributions of this work are as follows.

- We develop a scalable, simulation free framework for training EBMs by taking advantage of stochastic interpolants, score matching and noise contrastive estimation
- We demonstrate that both loss functions are crucial for effective model training, and are subsequently used to learn likelihoods of generated molecular conformations.
- We show that the learned likelihoods can be used to reweight conformations to match the Boltzmann distribution. To the best of our knowledge, this is the first method to recover the Boltzmann distribution without requiring exact likelihood computations.
- We further demonstrate that our model enables accurate estimation of free energy differences with orders-of-magnitude speedup.

### 2 Related Works

**Boltzmann Generators:** Boltzmann Generators have become an active and popular area of research since the publication of the initial work using invertible neural networks [4]. They have been used to sample the Boltzmann distributions of molecules [7, 8, 26–29] as well as lattice systems [7, 30–32]. Recent work [33] also introduces a more stable reweighting scheme that takes advantage of Jarzynski's equality to attain the equilibrium distribution. However, most of these methods have required input through system-specific featurizations such as internal coordinates, thereby hindering transferability. The emergence of CNFs and equivariant neural network architectures has enabled the

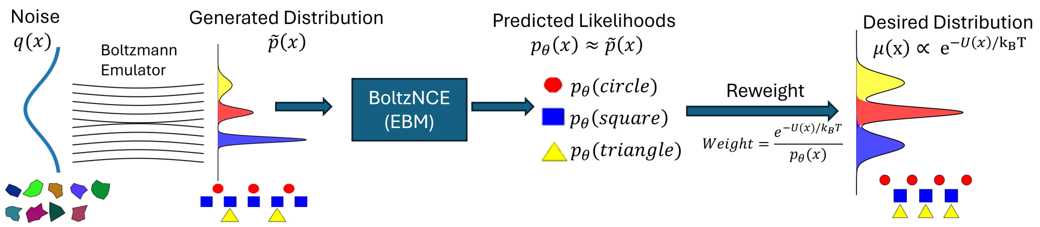

Figure 1: Method overview: Samples from a simple prior are transformed to a distribution of conformers/states by a Boltzmann Emulator. The generated samples are then reweighted with likelihoods estimated by the BoltzNCE model which is trained to approximate the generated distribution. After reweighting we achieve samples from the desired Boltzmann distribution.

development of Boltzmann Generators on Cartesian coordinates [5, 9]. Despite these advancements, transferability has so far only been demonstrated on small systems, such as dipeptides, primarily due to the computational limitations associated with likelihood evaluation at scale.

**Boltzmann Emulators:** Boltzmann Emulators, unlike Boltzmann Generators, are designed solely to produce high-quality samples without reweighting to the Boltzmann distribution. Because they are not required to be invertible, they can typically be applied to much larger systems. This flexibility also enables the use of a wider range of generative approaches, including diffusion models. Boltzmann Emulators have been employed to generate peptide ensembles [34], protein conformer distributions [35–38], small molecules [39–41], and coarse-grained protein structures [42, 43]. However, they are inherently limited by the data distribution they were trained on. As a result, they are generally unsuitable for generating unbiased samples from the Boltzmann distribution or for performing free energy calculations independently. In this work, we aim to leverage the strengths of Boltzmann Emulators and bridge the gap between Emulators and Generators using energy-based models (EBMs).

**Energy Based Models:** Energy-Based Models (EBMs) are particularly appealing in the physical sciences, as they describe density functions in a manner analogous to the Boltzmann distribution. This similarity enables the use of various techniques from statistical physics to compute thermodynamic properties of interest [28, 44]. Despite their promise, training EBMs remains a challenging task. However, recent advancements have introduced training objectives inspired by noise contrastive estimation [15, 17, 21, 44–46], contrastive learning [25, 47], and score matching [12, 48, 49]. Recent work [50] has also proposed an "energy-matching" objective to train a neural sampler on the Boltzmann distribution; however more work needs to be done to make this approach practical for molecules.

### 3 Background

#### 3.1 Boltzmann Generators

Boltzmann Generators (BG) utilize generative methods that sample conformers along with exact likelihoods so that the generated samples can be reweighted to the Boltzmann distribution. For instance, a BG model is trained to sample from a distribution $\tilde{p}(x)$ that is close to the Boltzmann distribution $\mu(x) \propto \exp(-U(x)/K_B T)$. Boltzmann generators are usually limited to the class of invertible methods due to the requirement of obtaining exact likelihoods.

Boltzmann generators can be used to obtain unbiased samples of the Boltzmann distribution by first sampling $x \sim \tilde{p}(x)$ with the exact likelihood and then reweighting with the corresponding importance weight given by $w(x) = \exp(\frac{-U(x)}{K_B T})/\tilde{p}(x)$. Leveraging these weights we can also approximate any observable, $O(x)$, under the Boltzmann distribution $\mu$ using self-normalized importance sampling:

$$\langle O \rangle_\mu = \mathbb{E}_{x \sim \tilde{p}(x)} [w(x)O(x)] \approx \frac{\sum_{i=1}^N w(x^i)O(x^i)}{\sum_{i=1}^N w(x^i)} \tag{1}$$

#### 3.2 Continuous Normalizing Flows

Normalizing flows are a class of generative models that transform samples from a simple prior distribution $x_1 \sim q(x)$ to samples of the generated distribution $x_0 \sim \tilde{p}(x)$ through a composition of invertible transforms.

Continuous Normalizing Flows (CNFs) are a continuous, time conditioned variant of normalizing flows that construct the invertible transformation on samples using the following ordinary differential equation

$$\frac{dx_t}{dt} = v_\theta(t, x_t) \tag{2}$$

where $v_\theta(t, x) : \mathbb{R}^n \times [0, 1] \to \mathbb{R}^n$ defines a time-dependent vector field and is parameterized by $\theta$. We can define a process that goes from time $t = 1$ to $t = 0$ that evolves $x_t$ according to $v_\theta$. Solving this initial value problem provides the transformation equation:

$$x_0 = x_1 + \int_1^0 v_\theta\big(t, x_t\big)\, dt \tag{3}$$

We can calculate the change in log density associated with the path integral described in Eq 3 through the following integral:

$$\log \tilde{p}(x_0) \;=\; \log q(x_1) \;-\; \int_1^0 \nabla \cdot\, v_\theta\big(t, x_t\big)\, dt \tag{4}$$

This likelihood integral involves computing the trace of the Jacobian along the vector field path. It require $O(DT)$ backpropogations where $D$ is the dimensionality of the data and $T$ is the number of integration timesteps. Therefore this approach is not scalable to large systems.

CNFs can be trained in a simulation-free manner using flow matching. For more details refer Section A.1.

#### 3.3 Stochastic Interpolants

Stochastic Interpolants are processes that turn noise sampled from a simple Gaussian prior $x_1 \sim \mathcal{N}(0, \mathbf{I})$ to data $x_0 \sim p_*(x)$. The time dependent process is as follows:

$$x_t = \alpha_t x_0 + \sigma_t x_1 \tag{5}$$

Here, $\alpha_t$ is a decreasing function of $t$ and $\sigma_t$ is a increasing function of $t$. The process is restricted on $t \in [0, 1]$ such that $x_t$ is exactly $x_1$ at $t = 1$ ($\alpha_1 = 0, \sigma_1 = 1$) and $x_0$ at $t = 0$ ($\alpha_0 = 1, \sigma_0 = 0$).

The sample $x_t$ under the stochastic interpolant evolves according to a vector field:

$$\frac{dx_t}{dt} = v(t, x_t), \tag{6}$$

where the vector field is given by the following conditional expectation:

$$v(t, x) = \dot{\alpha}_t \mathbb{E}\left[x_0 | x_t = x\right] + \dot{\sigma}_t \mathbb{E}\left[x_1 | x_t = x\right] \tag{7}$$

The score function, $s(t, x) = \nabla \log p_t(x)$, of the probability flow ODE associated with the interpolant is given by the following conditional expectation:

$$s(t, x) = \sigma_t^{-1} \mathbb{E}\left[x_1 | x_t = x\right] \tag{8}$$

Given a coupling of the prior and data distribution $C(x_0, x_1)$, we can learn the vector field and score function through the following objective functions:

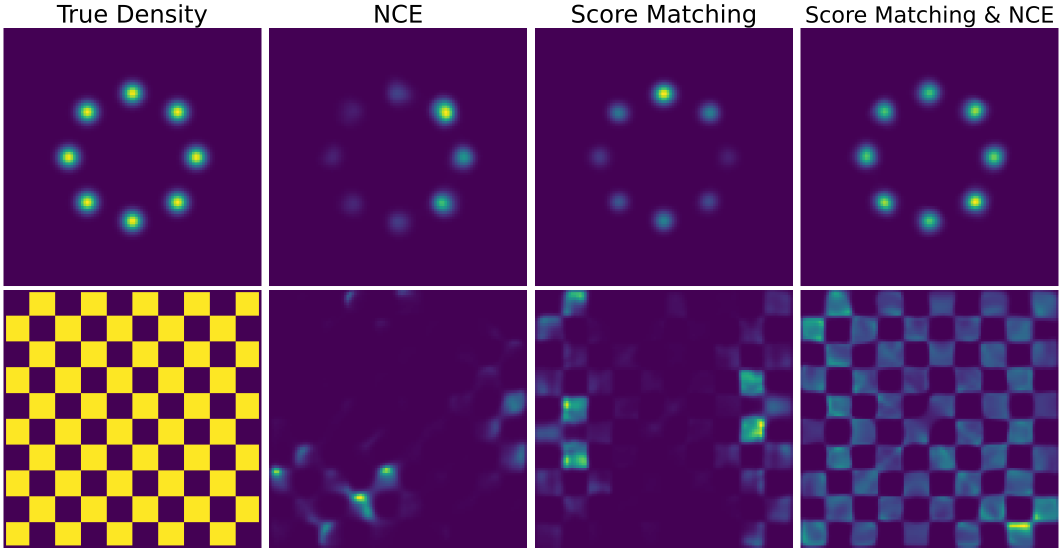

Figure 2: EBM density learnt on toy 2D systems - 8 Gaussians (above) and Checkerboard (below). The true density for the systems is shown on the left and the results for using different objective functions are labeled. It is clear that using both the NCE and score matching objectives (right) provides the best performance.

$$\mathcal{L}_v = \mathbb{E}_{t\sim\mathcal{U}(0,1),\,(x_0,x_1)\sim C(x_0,x_1)}\left[\|v_\theta(t,x_t) - \dot{\alpha}_t x_0 - \dot{\sigma}_t x_1\|^2\right] \tag{9}$$

$$\mathcal{L}_s = \mathbb{E}_{t\sim\mathcal{U}(0,1),\,(x_0,x_1)\sim C(x_0,x_1)}\left[\|\sigma_t s_\theta(t,x_t) + x_1\|^2\right] \tag{10}$$

The vector field objective (Eq. 9) can be further modified so that the model is trained to predict the final endpoint $x_0$ instead of the vector field. Through simple algebraic rearrangement, it can be shown that the objective function 9 is equivalent to the following endpoint objective:

$$\mathcal{L}_{EP} = \mathbb{E}_{t\sim\mathcal{U}(0,1),\,(x_0,x_1)\sim C(x_0,x_1)}\left[\|\frac{\dot{\alpha}_t\sigma_t - \alpha_t}{\sigma_t}(\hat{x}_0(t,x_t) - x_0)\|^2\right] \tag{11}$$

Where $\hat{x}_0(t,x_t)$ is the predicted endpoint by the neural network model. For more detail, refer section A.2. In this work we use both the vector field (Eq. 9) and endpoint (Eq. 11) objectives to train our CNF models.

### 4 Energy Based Model training with InfoNCE and Score Matching

Enery Based Models parametrize the density function as proportional to the exponential of a learned energy function $E_\theta(x)$ as follows:

$$p_\theta = \frac{\exp(E_\theta(x))}{Z_\theta}, \quad Z_\theta = \int \exp(E_\theta(x))dx \tag{12}$$

We can also make the density function time-dependant when the samples $x_t$ evolves according to a time process (e.g. with stochastic interpolants)

$$p_\theta(t,x_t) = \frac{\exp(E_\theta(t,x_t))}{Z_\theta(t)}, \quad Z_\theta(t) = \int \exp(E_\theta(t,x))dx \tag{13}$$

Given access to samples from a simple prior distribution $x_1 \sim \mathcal{N}(0,\mathbf{I})$, samples from a data distribution $x_0 \sim p_*(x)$ and a coupling function $C(x_0,x_1)$, we can obtain sample $x_t$ at time point $t$ with stochastic interpolants, as given by Eq.5.

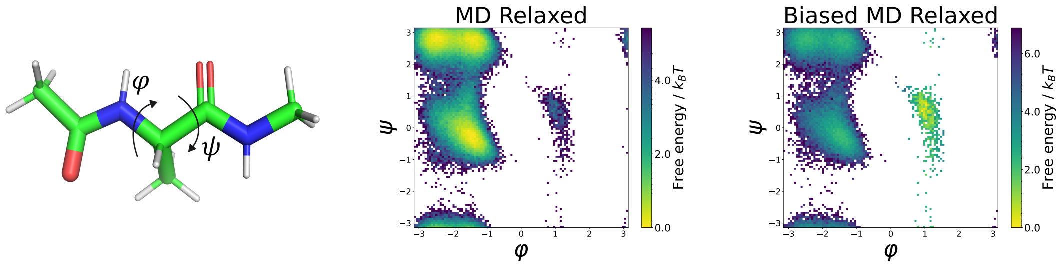

Figure 3: Alanine dipeptide (left) with dihedral angles labeled, Ramachandran plots of unbiased (center) and biased (right) datasets.

To maximize the likelihood of pair $(t, x_t)$ occurring under the distribution $p_\theta$ modeled by the energy based model, we minimize the negative log likelihood given by:

$$\mathcal{L}_{\text{NLL}} = \sum_{i=1}^{N} -\log \frac{\exp(E_\theta(t^i, x_t^i))}{\int \exp(E_\theta(t, x_t^i))dt} \tag{14}$$

The intractable integral in the denominator can be approximated by appropriately sampling a set of *negative* time points $\{\tilde{t}^i\}$ yielding the InfoNCE loss given by:

$$\mathcal{L}_{\text{InfoNCE}} = \sum_{i=1}^{N} -\log \frac{\exp(E_\theta(t^i, x_t^i))}{\sum_{t' \in \{\tilde{t}^i\} \cup t^i} \exp(E_\theta(t', x_t^i))} \tag{15}$$

Note that this objective function is *simulation-free* as it only requires sampling of negative time points. Furthermore, the gradient of the energy function is also the score of the model's density $\nabla \log p_\theta(t, x_t) = \nabla E_\theta(t, x_t)$, therefore we can use the well defined score matching objective (Eq. 10) associated with stochastic interpolants as an additional objective to train the EBM as follows:

$$\mathcal{L}_{\text{SM}} = \mathbb{E}_{t \sim \mathcal{U}(0,1),\,(x_0,x_1) \sim C(x_0,x_1)} \left[ \|\sigma_t \nabla E_\theta(t, x_t) + x_1\|^2 \right] \tag{16}$$

Where $C(x_0, x_1)$ is a coupling function, and $x_t$ is computed using Eq. 5. We show that both objectives are important for optimal model performance on toy 2D systems in Figure 2 and therefore use both loss functions for subsequent experiments on molecular systems.

## 5 Methods

### 5.1 Overview

Our method is designed to calculate free energy values and attain the Boltzmann distribution in a scalable manner. We first train Boltzmann Emulators on a dataset of conformers to learn the distribution $\tilde{p}(x)$. An EBM is then trained on conformers sampled from the emulator $x \sim \tilde{p}(x)$ to approximate the learnt distribution $p_\theta(x) \approx \tilde{p}(x)$ (up to a normalization constant). Specifically, the EBM is trained using stochastic interpolants, therefore the density function at time point "0" approximates the desired distribution $p_\theta(t = 0, x) \approx \tilde{p}(x)$. The generated samples are then reweighted to the Boltzmann distribution with the (unnormalized) importance weights being a ratio of Boltzmann factors and EBM densities $w(x) = \frac{\exp(-U(x)/K_B T)}{p_\theta(0,x)}$. An overview of our method is shown in Figure 1.

### 5.2 Datasets

For our experiments, we use the well-studied alanine dipeptide molecule. The dataset employed is the same as that used in [5]. Briefly, it is generated via molecular dynamics (MD) simulations using the classical force field Amber ff99SBildn, followed by relaxation with the semi-empirical GFN-xTB force field. We utilize the dataset in two variants: one in its original form (referred to as unbiased), and another in which the positive $\varphi$ metastable state (see Ramachandran plots, Figures 3) is oversampled to ensure equal representation of both positive and negative states (referred to as biased). For more details refer Section E.1.

#### 5.3 Training and inference algorithms

Both the Boltzmann Emulator and EBMs are trained taking advantage of stochastic interpolants where samples $(x_1, x_0)$ are coupled through mini-batch optimal transport. In practice, we use the Hungarian algorithm for OT coupling as it provides the best scalability with batch size.

Boltzmann Emulator models are trained using either the vector field or the endpoint loss functions specified by the stochastic interpolant (Eqs 9, 11). With the endpoint parameterization, the vector field integrated for sampling is given by:

$$v_\theta(t, x) = \sigma_t^{-1}(\dot{\sigma_t} x_t + (\dot{\alpha_t}\sigma_t - \alpha_t)\hat{x}_0) \tag{17}$$

where $\hat{x}_0$ is the predicted endpoint at time-point $t$. Note that the given vector field diverges at $t = 0$ ($\sigma_0 = 0$). In practice, we integrate endpoint models only till $t = 1e - 3$ to account for this.

The EBMs are trained using InfoNCE (Eq. 15) and score matching (Eq. 10) objectives. In practice, we found that using a single negative time point per sample was sufficient for effective training. These negative time points are sampled from a narrow Gaussian distribution centered around the corresponding positive time point, $t' \sim \mathcal{N}(t, 0.025)$ which yields informative negatives for training the model.

For more details on model training and inference, refer sections B, E.5, E.7 in the appendix.

#### 5.4 Model architectures

For both models, the data is featurized using the same atom typing scheme as described in [5]. Briefly, all atoms are kept distinguishable, except for hydrogen atoms bonded to the same carbon or nitrogen. The molecular structures are passed as fully connected graphs, and both models operate directly on the Cartesian coordinates of the atoms.

Boltzmann Emulators are parameterized with a SE(3) - equivariant graph neural network that leverages Geometric Vector Perceptrons (GVPs) [51]. Briefly, GVP maintains a set of equivariant vector and scalar features per node that are updated in an SE-(3) equivarant/invariant manner through graph convolutions. We utilize this architecture as it has been shown to have improved performance over Equivariant Graph Neural Networks (EGNNs) [52] in molecular design tasks [53].

EBMs are implemented using the Graphormer [54] architecture, which has demonstrated state-of-the-art performance in molecular property prediction tasks. Graphormers function similarly to standard Transformers, with the key difference being the incorporation of an attention bias derived from graph-specific features. In 3D-Graphormers, this attention bias is computed by passing a Euclidean distance matrix through a Multi-Layer Perceptron (MLP).

For more details on model architectures and hyperparameters, refer sections C, E.3 in the appendix.

### 6 Results

GVP-based Boltzmann Emulators trained using the vector field (referred to as GVP-VF) and endpoint objectives (referred to as GVP-EP) are compared in Section 6.1. These emulators are evaluated on the unbiased dataset.

In Section 6.2, the emulators are trained on the biased dataset, and their performance as Boltzmann Generators for generating the semi-empirical distribution of alanine dipeptide induced by the GFN-xTB forcefield is assessed. The EBMs trained on the GVP-based emulators are also evaluated and are referred to as BoltzNCE-VF/BoltzNCE-EP. Free energy differences between the positive and negative $\varphi$ metastable states are computed as it is the slowest process (Figure 3).

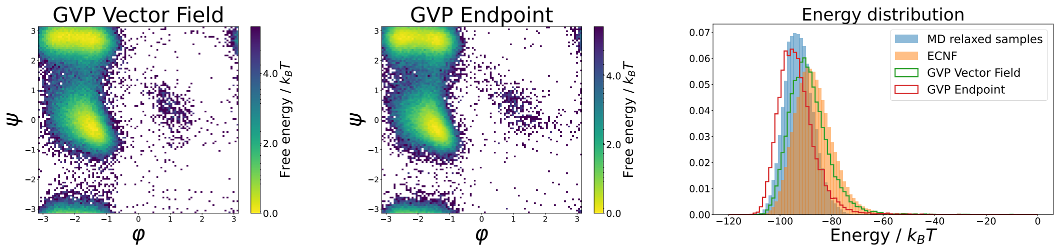

Figure 4: Ramachandran plots of samples generated by the GVP Vector field model (left), GVP Endpoint model (center) and energy distribution of generated samples (right).

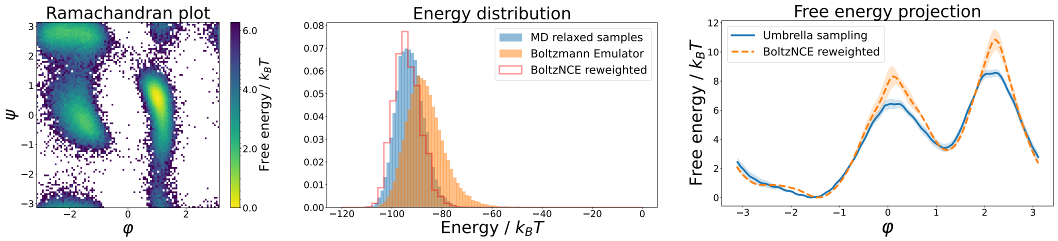

Figure 5: BoltzNCE results for alanine dipeptide trained on the biased dataset. The GVP vector field model is used as the Boltzmann Emulator. Ramachandran plot of generated samples is shown on the left, energy histogram along with BoltzNCE reweighting on the center and calculated free energy surfaces for the angle $\varphi$ on the right.

In both sections we benchmark our models against the Equivariant Continuous Normalizing Flow (ECNF) model from [5] trained on respective datasets. For more details on the metrics used, refer section D in the appendix.

#### 6.1 GVP models are good Boltzmann Emulators

Inference results for the Boltzmann Emulators are presented in Table 1 and Figure 4. The Energy ($\mathcal{E}$) and Torsion angle ($\mathbb{T}$) Wasserstein-2 ($W_2$) distances quantify the discrepancy between the distributions of generated conformers and those in the dataset with respect to energy and torsion angles respectively. The results show that while the $\mathbb{T}$-$W_2$ distance remains relatively consistent across all methods, the GVP models capture the dataset's energy distribution better, with the Endpoint model showing the best performance (Figure 4) indicating that it is a very good Boltzmann Emulator on this dataset.

The ECNF and GVP-VF models are comparable on the Negative Log Likelihood (NLL) metric, whereas the GVP-EP model yields the worst values. It is important to note, however, that the endpoint vector field (Eq. 17) diverges at time-point 0. Consequently, the likelihoods for the GVP-EP model were evaluated starting from a later time point $t = 1e - 3$. Furthermore, the divergence at $t \to 0$ can lead to inaccurate likelihood estimates due to instability in the ODE integration. The standard deviation of NLL values within each run is also reported, and the large variance observed for the GVP-EP model further highlights the potential unreliability of its likelihood computations. As we will see in the next section, this inaccuracy in likelihood calculations also make the GVP-EP model unsuitable Boltzmann Generators despite being excellent invertible Emulators.

#### 6.2 Reweighting Emulators with BoltzNCE yields accurate free energy estimates

Free energy differences computed by all models across five runs are reported in Table 2. We also reproduce the ECNF model and report results for the same. The ECNF, GVP-VF, and GVP-EP models estimate likelihoods using the Jacobian trace integral and serve as Boltzmann Generators. In contrast, the BoltzNCE models are EBMs trained on conformers generated by the GVP models (Boltzmann Emulators) and provide direct access to predicted likelihoods of generated conformers.

Focusing on the Boltzmann Generator models, we observe that the GVP models produce less accurate estimates of free energy difference despite being comparable or better Boltzmann Emulators. This

Table 1: Comparison of NLL and $W_2$ metrics of Boltzmann Emulators across 5 runs

| Method | $\mathcal{E}$-$W_2$ | $\mathbb{T}$-$W_2$ | NLL | NLL std |
|---|---|---|---|---|
| ECNF | 5.84 ± 0.04 | 0.27 ± 0.01 | **-125.53 ± 0.10** | **5.09 ± 0.09** |
| GVP Vector Field | 3.76 ± 0.08 | 0.27 ± 0.02 | -125.42 ± 0.15 | 6.92 ± 0.62 |
| GVP Endpoint | **1.76 ± 0.11** | **0.26 ± 0.02** | -92.04 ± 3.24 | 175.12 ± 35.51 |

Table 2: Dimensionless free energy differences calculated for the slowest transition of alanine dipeptide along the $\varphi$ angle by several methods. Errors shown across 5 runs. Free energy difference values for Umbrella sampling and ECNF taken from [5].

| Method | $\Delta$ F / $k_B T$ | Inference-time (h) | Train-time(h) | Jac-trace integral |
|---|---|---|---|---|
| Umbrella Sampling | 4.10 $\pm$ 0.26 | - | - | - |
| ECNF[5] | **4.09 $\pm$ 0.05** | 9.366 | **3.85** | ✓ |
| ECNF - reproduced | 4.07 $\pm$ 0.23 | 9.366 | **3.85** | ✓ |
| GVP Vector field | 4.38 $\pm$ 0.67 | 18.42 | 4.42 | ✓ |
| GVP Endpoint | 4.89 $\pm$ 2.61 | 26.24 | 4.42 | ✓ |
| BoltzNCE Vector field | 4.08 $\pm$ 0.13 | **0.09** | 12.22 | ✗ |
| BoltzNCE Endpoint | 4.14 $\pm$ 0.94 | 0.164 | 12.22 | ✗ |

inaccuracy may stem from unreliable likelihood estimates produced during ODE integration. The instability in accurate likelihood estimation in continuous normalizing flows (CNFs) requires the model to behave consistently under the Jacobian trace integral. Various factors, including model architecture, training protocol, etc, can affect the numerical stability of this integral. As a result, Boltzmann Generator models face additional design constraints to ensure stability and reliability in likelihood estimation.

In contrast, the BoltzNCE models yield more accurate estimates of the free energy difference compared to the GVP-based Boltzmann Generators. This indicates that the likelihoods predicted by BoltzNCE may be more reliable than those obtained via the Jacobian trace integral in these generators. On comparison, the BoltzNCE-EP model exhibits higher variance compared to the free energy estimates from the BoltzNCE-VF models. Representative energy histogram and free energy surfaces along the slowest transition ($\varphi$ dihedral angle) for the BoltzNCE-VF model is shown in Figure 5. For energy histograms and free energy projections of other methods, refer section F.

The inference time costs includes the time to generate and estimate likelihoods for $1 * 10^6$ conformers of alanine dipeptide. BoltzNCE provides an overwhelming inference time advantage over the Boltzmann Generator by multiple orders-of-magnitude. This is especially advantageous as it is often desirable to perform evaluation across multiple sets of samples to get more confident estimates. It is important to note, however, that BoltzNCE has an upfront cost of training the EBM associated with it.

### 7 Discussion

In this work, we propose a novel, scalable, and simulation-free training framework for energy-based models that leverages stochastic interpolants, InfoNCE, and score matching. We demonstrate that both the InfoNCE and score matching objectives play a complementary role in enhancing model performance. Our training approach is applied to learn the density function of conformers sampled from a Boltzmann Emulator, thereby eliminating the need for expensive Jacobian trace calculations for reweighting, resulting in orders-of-magnitude speedup. Furthermore, experiments conducted on alanine dipeptide indicate that in certain cases BoltzNCE is even capable of providing more accurate estimates than ODE integration of the divergence operator. This framework, therefore, effectively bridges the gap between Boltzmann Emulators and Generators, and removes the requirement for invertible architectures in Boltzmann Generator design or costly Jacobian trace calculations.

### 8 Limitations and Future Work

The present work is limited to the alanine dipeptide molecular system. However, although not explicitly demonstrated, the proposed framework is potentially transferable across multiple molecular systems and also scalable to larger molecular systems. The accuracy of the method needs to be further tested in these scenarios.

Training the energy-based model requires applying the score matching loss to its gradients, which increases compute requirements beyond typical levels for neural networks training. Additionally, since the likelihoods estimated by the EBM are approximate, a degree of mismatch between the samples and their predicted likelihoods is inevitable.

Although the current work is limited to a molecular setting, we believe the proposed EBM training framework could be broadly applicable in other domains where energy-based models are useful, such as robotics.

### 9 Acknowledgments

We thank Leon Klein for making the code and data for his original Boltzmann Generator and Equivariant Flow Matching methods readily available.

This work is funded through R35GM140753 from the National Institute of General Medical Sciences. The content is solely the responsibility of the authors and does not necessarily represent the official views of the National Institute of General Medical Sciences or the National Institutes of Health.

### A Vector field training objectives

#### A.1 Training with Conditional Flow Matching

CNFs can be trained in a *simulation-free* manner through flow matching. One can formulate the flow matching objective using:

$$\mathcal{L}_{\mathrm{FM}}(\theta) = \mathbb{E}_{t\sim\mathcal{U}(0,1),\, x\sim p_t(x)} ||v_\theta(t,x) - v_t(x)||_2^2 \tag{18}$$

where $v_t$ is the target vector field and $v_\theta(t,x)$ is the learned vector field. The conditional flow matching objective [55], however, utilizes a conditioning variable $z$ to make it more tractable

$$\mathcal{L}_{\mathrm{CFM}}(\theta) = \mathbb{E}_{t\sim\mathcal{U}(0,1),\, x\sim p_t(x|z)} ||v_\theta(t,x) \; - \; u_t(x \mid z)||_2^2 \tag{19}$$

where $u_t(x \mid z)$ is the conditional vector field. There are several different ways to construct the conditional vector field and probability path. For example, a simple parametrization used by [9] for training Boltzmann Generators is as follows:

$$z = (x_0, x_1), \quad p(z) = \pi(x_0, x_1) \tag{20}$$

$$v_t(x \mid z) = x_1 - x_0 \quad p_t(x \mid z) = \mathcal{N}(x \mid t.x_1 + (1-t).x_0, \sigma^2) \tag{21}$$

where $\pi$ is the 2-Wasserstein optimal transport plan between the prior $q(x_0)$ and data distribution $\mu(x_1)$. For further details please refer [9].

#### A.2 Proof of Endpoint Parametrization Eq. 11

Stochastic interpolants anneal between $x_1 \sim \mathcal{N}(0, \mathbf{I})$ and $x_0 \sim p_*(x)$ with:

$$x_t = \alpha_t x_0 + \sigma_t x_1 \tag{22}$$

solving for $x_1$:

$$x_1 = \frac{x_t - \alpha_t x_0}{\sigma_t} \tag{23}$$

$x_t$ evolves according to the vector field given by the conditional expectation:

$$v(t,x) = \dot{\alpha}_t \mathbb{E}\left[x_0 | x_t = x\right] + \dot{\sigma}_t \mathbb{E}\left[x_1 | x_t = x\right] \tag{24}$$

Substituting 23 in 24 we get:

$$v(t,x) = \dot{\alpha}_t \mathbb{E}\left[x_0 | x_t = x\right] + \frac{\dot{\sigma}_t (x - \alpha_t \mathbb{E}\left[x_0 | x_t = x\right])}{\sigma_t} \tag{25}$$

$$v(t,x) = \sigma_t^{-1}(\dot{\sigma}_t x + (\dot{\alpha}_t \sigma_t - \alpha_t)\mathbb{E}\left[x_0 | x_t = x\right]) \tag{26}$$

Similarly, the model estimate of the vector field is given by:

$$v_\theta(t,x) = \sigma_t^{-1}(\dot{\sigma}_t x + (\dot{\alpha}_t \sigma_t - \alpha_t)\hat{x}_0(t,x)) \tag{27}$$

Where $\hat{x}_0(t, x_t)$ is the predicted endpoint by the model. The objective is then given by:

$$\mathcal{L}_{EP} = \int_0^T \|v_\theta(t, x_t) - v(t, x_t)\|^2 dt \tag{28}$$

$$\mathcal{L}_{EP} = \int_0^T \mathbb{E}\left[ \| \frac{\dot{\alpha}_t \sigma_t - \alpha_t}{\sigma_t} (\hat{x}_0(t, x_t) - x_0) \|^2 \right] dt \tag{29}$$

## B EBM training algorithm

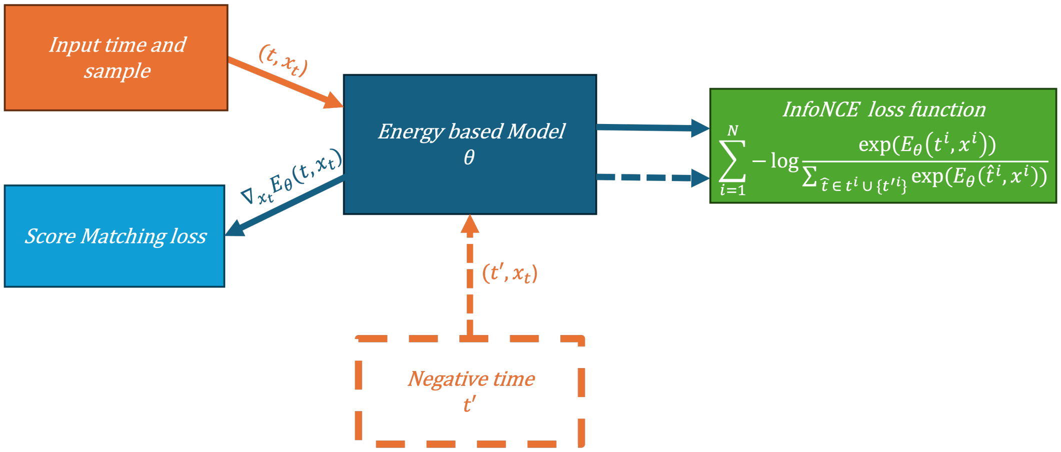

Figure 6: Energy Based Model training workflow

A diagramatic representation of the method used for training the energy based model is shown in Figure 6. The model takes a sample $x$ and time point $t$ as input and outputs predicted energy $E_\theta(t, x)$. The gradient of the output w.r.t to the sample $\nabla_x E_\theta(t, x)$ is used for the score matching loss. The same sample is also passed with negative time points $\{t'\}$, and the predicted energies $E_\theta(t', x)$ is used along with the previously output energies $E_\theta(t, x)$ for the InfoNCE loss.

We also provide a pseudocode block for training the energy based model with stochastic interpolants, InfoNCE, and score matching in algorithm block 1.

**Algorithm 1:** Training EBM with stochastic interpolants, InfoNCE, and score matching

**Input:** Energy-Based model $\theta$, samples from prior $X_1$, dataset samples $X_0$, interpolant functions $\alpha_t, \sigma_t$

**for** *epoch* $\leftarrow 1$ **to** *epoch*$_{\max}$ **do**
- **for** *batch* $(x_1, x_0)$ *in* $(X_1, X_0)$ **do**
  - $(x_1, x_0) \leftarrow \texttt{mini-batch OT}(x_0, x_1)$
  - **sample** $t \sim \mathcal{U}(0, 1)$
  - $x_t \leftarrow \alpha_t x_1 + \sigma_t x_0$
  - $\mathcal{L}_{SM} \leftarrow \frac{1}{N} \sum_{n=1}^{N} |\sigma_t \nabla E_\theta(t^n, x_t^n) + x_1^n|^2$
  - **sample** $t' \sim \mathcal{N}(t, 0.025)$
  - $\mathcal{L}_{\text{InfoNCE}} \leftarrow \frac{1}{N} \sum_{n=1}^{N} -\log \frac{\exp(E_\theta(t^n, x_t^n)}{\exp(E_\theta(t^n, x_t^n) + \exp(E_\theta(t'^n, x_t^n)}$
  - $\mathcal{L} \leftarrow \mathcal{L}_{\text{SM}} + \mathcal{L}_{\text{InfoNCE}}$
  - $\theta \leftarrow \texttt{Update}(\theta, \nabla_\theta \mathcal{L})$

**Output:** Updated model parameters $\theta$

## C Model Architecture

### C.1 GVP Convolutions

For our models, we use a modified version of the GVP which has been shown to increase performance as describe in [56]. The message passing step is constructed by applying the GVP message passing operation defined in [57].

$$(m_{i \to j}^{(s)}, m_{i \to j}^{(v)}) = \psi_M \left( [h_i^{(l)} : d_{ij}^{(l)}],\ v_i : \left[ \frac{x_i^{(l)} - x_j^{(l)}}{d_{ij}^{(l)}} \right] \right) \tag{30}$$

Here $m_{i\to j}^{(v)}$ and $m_{i\to j}^{(s)}$ are the vector and scalar messages between nodes $i$, $j$. $h_i$,$d_{ij}$ are the scalar features, edge features and a radial basis embedding respectively, while $x$ represents the coordinates of the node. For the detailed Node Position Update and Node Feature Update operations, refer to Appendix C of [56].

#### C.2 Graphormer Operations

Graphformers are neural network architectures where layer-wise GNN components are nested alongside typical transformer blocks. [58] For our EBMs, we follow the implementation of the Graphformer with one minor modification. For the original Graphformer, each attention head is calculated as :

$$\text{head} = \text{softmax}\Big(\frac{QK^{\mathsf{T}}}{\sqrt{d}} + B\Big)\, V \tag{31}$$

where $B$ is a learnable bias matrix. In our implementation, $B$ is calculated by passing the graph's euclidean distance matrix through an MLP.

### D Metrics

#### D.1 NLL

To calculate the NLL of the holdout conformers, we take (4) and evaluate the ODE in the reverse direction for a given sample. This provides the NLL of the sample. NLL values are reported over batches of $1 * 10^3$ samples.

#### D.2 Energy - W2

In order to quantify the difference in energy distributions between generated molecules and MD relaxed samples, we calculate the Wasserstein-2 distance between the two distributions. This can be intuitively thought of as the cost of transforming one distribution to another using optimal transport. Mathematically, we solve the optimization process with the loss:

$$\mathcal{E}\text{-}W_2 = \left(\inf_{\pi} \int c(x,y)^2 \, d\pi(x,y)\right)^{\frac{1}{2}} \tag{32}$$

where $\pi(x,y)$ represents a coupling between two pairs $(x,y)$ and $c(x,y)$ is the euclidean distance. We use the Python Optimal Transport package in our implementation [59]. $\mathcal{E}$-$W_2$ values are reported over batches of $1 * 10^5$ samples.

#### D.3 Angle - W2

Similar to the $\mathcal{E}$-$W_2$ metric, we seek to quantify the differences in the distributions of dihedral angles generated and those from MD relaxed samples. Here, following the convention defined in [33] we define the optimal transport in torsional angle space as:

$$\mathbb{T}\text{-}W_2 = \left(\inf_{\pi} \int c(x,y)^2 \, d\pi(x,y)\right)^{\frac{1}{2}} \tag{33}$$

where $\pi(x,y)$ represents a coupling between two pairs $(x,y)$. The cost metric on torsional space is defined as:

$$c(x,y) = \left(\sum_{i=1}^{2s} \big((x_i - y_i)\%\pi\big)^2\right)^{\frac{1}{2}} \tag{34}$$

where $(x,y) \in [-\pi,\pi)^{2s}$

Similar to Energy-W2 calculations, we use the Python Optimal Transport package for implementation [59]. $\mathbb{T}$-$W_2$ values are reported over batches of $1 * 10^5$ samples.

#### D.4 Free energy difference

Free energy differences are computed between the positive and negative metastable states of the $\varphi$ dihedral angle. The positive state is defined as the region between 0 and 2, while the negative state encompasses the remaining range. The free energy associated with each state is estimated by taking the negative logarithm of the reweighted population count within that state.

The code for calculating the free energy difference is as follows:

```
left = 0.
right = 2

hist, edges = np.histogram(phi, bins=100, density=True, weights=weights)
centers = 0.5*(edges[1:] + edges[:-1])
centers_pos = (centers > left) & (centers < right)

free_energy_difference = -np.log(hist[centers_pos].sum()/
hist[~centers_pos].sum())
```

Where *phi* is a numpy array containing the $\varphi$ angles of the generated dataset ($\varphi \in (-\pi, \pi]$) and *weights* is an array containing the importance weight associated with it.

#### D.5 Inference times

Inference time for free energy estimation is measured over $1 * 10^6$ samples. Specifically, we use a batch size of 500 and generate 200 batches of conformers. During sample generation, Boltzmann Generators also computes the Jacobian trace. All run times are recorded on NVIDIA L40 GPUs, and the reported values represent the mean of five independent runs.

### E Technical Details

#### E.1 Dataset Biasing

Since transitioning between the negative and positive $\varphi$ is the slowest process, with the positive $\varphi$ state being less probable, we follow the convention of [9, 5] and use a version of the dataset with bias to achieve nearly equal density in both states, which helps in obtaining a more accurate estimation of free energy. To achieve the biased distribution, weights based on the von Mises distribution, $f_{vM}$, are incorporated and computed along the $\varphi$ dihedral angle as

$$\omega(\varphi) = 150 \cdot f_{\text{vM}}\big(\varphi \mid \mu = 1, \kappa = 10\big) + 1 \tag{35}$$

For the biased dataset, samples are then drawn based on the weighted distribution.

#### E.2 Correcting for chirality

Since SE(3) equivariant neural networks are invariant to mirroring, the Emulator models tend to generate samples from both chiral state. To account for this, we fix chirality *post-hoc* following the convention set by [5, 9].

#### E.3 Model hyperparameters

Each GVP-Boltzmann Emulator model employs one message-passing GVP and one update GVP, each built from five hidden layers with vector-gating layers. Within every GVP, the hidden representation comprises 64 scalar features and 16 vector features.

Graphormer-based potential model were instantiated with a 256-dimensional node embedding and a matching 256-unit feed-forward inner layer in each transformer block with a total of 8 layers. Self-attention is employed with 32 heads over these embeddings,and inter-atomic distances are encoded via 50 gaussian basis kernels.

#### E.4 Endpoint training weights

The Endpoint loss function for training the Boltzmann Emulator is given by:

$$\mathcal{L}_{EP} = \mathbb{E}_{t\sim\mathcal{U}(0,1),\,(x_0,x_1)\sim C(x_0,x_1)}\left[\|\frac{\dot{\alpha}_t\sigma_t - \alpha_t}{\sigma_t}(\hat{x_0}(t,x_t) - x_0)\|^2\right] \quad (36)$$

Note that, the coefficients $\frac{\dot{\alpha}_t\sigma_t - \alpha_t}{\sigma_t}$ become divergent near $t \to 0$ as $\sigma_0 = 0$. Therefore, in practice, we threshold the min and the max value of these coefficients as follows:

$$t_w = \min(\max(0.005, |\frac{\dot{\alpha}_t\sigma_t - \alpha_t}{\sigma_t}|), 100) \quad (37)$$

And optimize the following objective:

$$\mathcal{L}_{EPmod} = \mathbb{E}_{t\sim\mathcal{U}(0,1),\,(x_0,x_1)\sim C(x_0,x_1)}\left[t_w\|\hat{x}_0(t,x_t) - x_0\|^2\right] \quad (38)$$

#### E.5 Training protocols

Models were trained for 1,000 epochs using the Adam optimizer with a learning rate of 0.001 and a batch size of 512. A learning rate scheduler was employed to reduce the rate by a factor of 2 after 20 consecutive epochs without improvement, down to a minimum of $1e^{-5}$. An Exponential Moving Average (EMA) with $\beta$ = 0.999 was applied to the model and updated every 10 iterations. Mini-batch optimal transport is computed using the scipy `linear_sum_assignment` function [60]. All models are trained on NVIDIA L40 GPUs with a batch size of 512.

#### E.6 Interpolant Formulation

We specify the interpolant process following the design choices explored in [24]. The Emulator models are trained with linear interpolants while the energy based models use trigonometric interpolants. Both of which satisfy the constraints to generate an unbiased interpolation process.

$$Linear: \alpha_t = 1 - t, \qquad \sigma_t = t \quad (39)$$

$$\text{Trignometric: } \alpha_t = cos(\frac{1}{2}\pi t), \qquad \sigma_t = sin(\frac{1}{2}\pi t) \quad (40)$$

Trigonometric interpolants are called general vector preserving interpolants (GVP) in [24]. However, we change the naming of this notation to avoid confusion with geometric vector perceptrons (GVP), which are repeatedly discussed in our paper.

#### E.7 Integration scheme

All models were integrated with the adaptive step size DOPRI5 solver implemented in the Torchdiffeq package [61]. The tolerance values were set to $\texttt{atol} = 1e^{-5}$, and $\texttt{rtol} = 1e^{-5}$. For vector field models, each integral is evaluated from 1 to 0, while endpoint models are evaluated from 1 to $1e^{-3}$ in order to avoid the numerical instability that occurs with endpoint parametrization at time $t = 0$

### F Additional Results

Energy histograms and free energy projections for GVP Vector Field, GVP Endpoint, and BoltzNCE Endpoint methods are show in Figure 7. The free energy values and energy histograms match up best with the BoltzNCE Endpoint method.

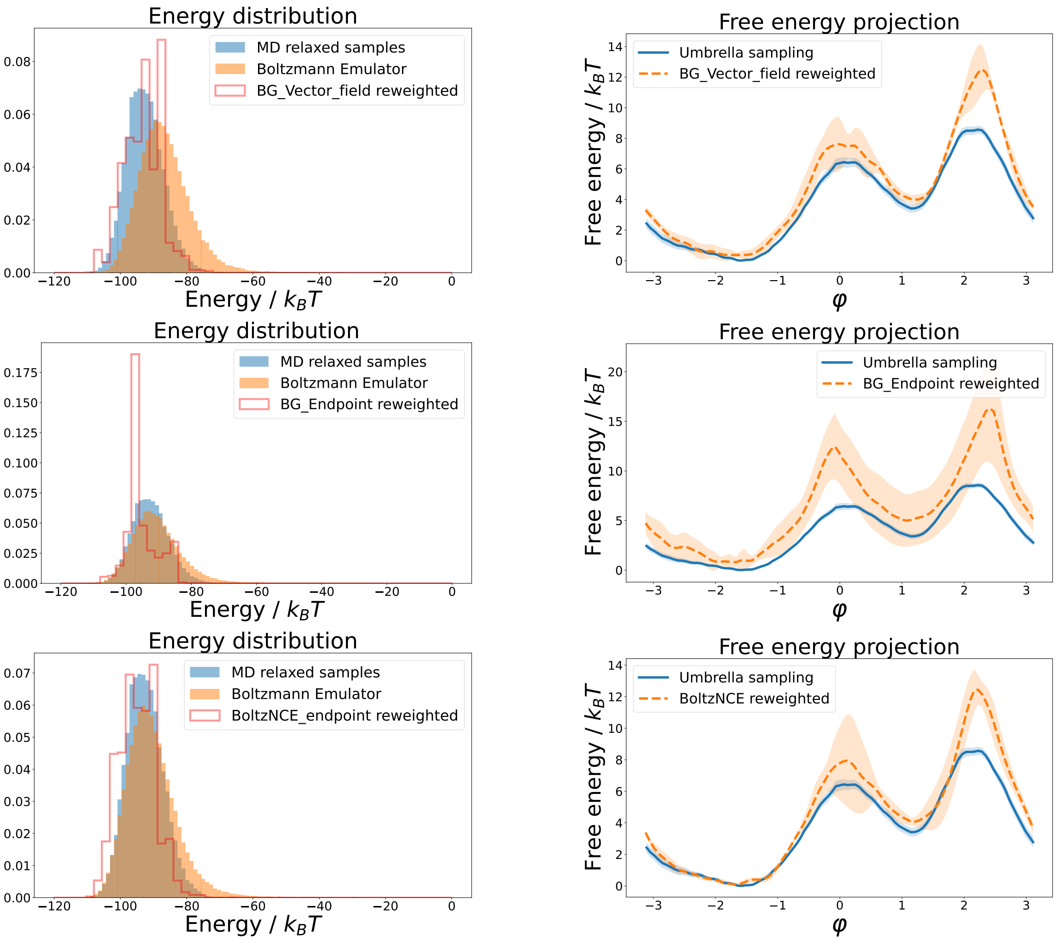

Figure 7: Energy histograms and free energy projections with confidence intervals for the GVP-Vector Field (**top**), GVP-Endpoint (**center**) and BoltzNCE-Endpoint (**bottom**) models.
